# Analysis of combat casualties admitted to the emergency department during the negotiation of the comprehensive Colombian process of peace

**DOI:** 10.25100/cm.v43i4.3389

**Published:** 2017-12-30

**Authors:** Carlos A Ordoñez, Ramiro Manzano Nunez, Michael W Parra, Juan Pablo Herrera, Maria Paula Naranjo, Sara Sofia Escobar, Marisol Badiel, Monica Morales, Cecibel Cevallos, Juan G Bayona, Alvaro Ignacio Sanchez, Juan Carlos Puyana, Alberto F García

**Affiliations:** 1 Division of Trauma and Acute Care Surgery. Fundación Valle del Lili. Cali, Colombia; 2 Universidad del Valle, Cali, Colombia; 3 Clinical Research Center. Fundación Valle del Lili. Cali, Colombia; 4 Broward Health Medical Center. Florida, USA; 5 Department of Surgery, Brigham & Women's Hospital. Boston, Massachusetts. USA; 6 University of Pittsburgh. Pittsburgh, Pennsylvania. USA

**Keywords:** Military personnel, wounds and injuries, critical care, critical care outcomes, warfare, policia militar, heridas y lesiones, cuidado critico, resultados en cuidado critico, guerra

## Abstract

**Aim:**

Our objective was to describe the variations in casualties admitted to the emergency department during the period of the negotiation of the comprehensive peace agreement in Colombia between 2011 and 2016.

**Methods:**

A retrospective study of all hostile military casualties managed at a regional Level I trauma center from January 2011 to December 2016. Patients were subsequently divided into two groups: those seen before the declaration of the process of peace truce (November 2012) and those after (negotiation period). Variables were compared with respect to periods

**Results:**

A total of 448 hostile casualties were registered. There was a gradual decline in the number of admissions to the emergency department during the negotiation period. The number of soldiers suffering blast and rifle injuries also decreased over this period. In 2012 there were nearly 150 hostile casualties' admissions to the ER. This number decreased to 84, 63, 32 and 6 in 2013, 2014, 2015 and 2016 respectively. Both, the proportion of patients with an ISS ≥9 and admitted to the intensive care unit were significantly higher in the period before peace negotiation. From August to December/2016 no admissions of war casualties were registered.

**Conclusion:**

We describe a series of soldiers wounded in combat that were admitted to the emergency department before and during the negotiation of the Colombian process of peace. Overall, we found a trend toward a decrease in the number of casualties admitted to the emergency department possibly in part, as a result of the period of peace negotiation.

## Resumen

**Objetivo:**

Describir las variaciones en los soldados heridos en combate admitidos al departamento de emergencias durante el periodo de negociación del proceso de paz colombiano entre el 2011 y el 2016.

**Métodos:**

Estudio retrospectivo de todos los soldados heridos en combate que fueron manejados en un centro de trauma desde Enero del 2011 a Diciembre del 2016. Los pacientes se dividieron en dos grupos: aquellos que ingresaron al departamento de emergencias antes de la tregua del proceso de paz (Noviembre 2012) y aquellos que ingresaron durante la negociación. Los grupos se compararon con respecto a los periodos de tiempo.

**Resultados:**

Un total de 448 soldados heridos en combate fueron incluidos. Hubo una disminución gradual en el número de admisiones durante el periodo de negociación. Además, el número de soldados que sufrieron heridas por explosiones y fusiles disminuyó durante este periodo. En el 2012 se registraron 150 soldados heridos en combate. Este número disminuyó a 84, 63, 32 y 6 en los años 2013, 2014, 2015 y 2016 respectivamente. La proporción de pacientes con un ISS ≥9 y la proporción de admitidos a la unidad de cuidado intensivo fueron significativamente mayores en el periodo antes de la negociación. Desde Agosto a Diciembre/2016 no se registraron admisiones.

**Conclusión:**

Este estudio describe una disminución gradual en el número de soldados heridos en combate admitidos al departamento de emergencia en un periodo de 6 años. Este fenómeno pudo deberse al periodo de negociación del proceso de paz.

## Introduction

After decades of armed conflict, in 2012 the Colombian Government agreed to begin long-awaited conversations with the left-wing guerrillas (FARC), with an eye to ending the widespread rural and urban violence that has plagued the country for decades. The internal armed conflict in Colombia has been characterized by a human security crisis of extraordinary dimensions. Over the past 50 years, there have been approximately 92.946 victims of hostile actions ^1^, and 39,000 violent deaths have occurred due to armed conflict since 1988 ^2^.

From a global perspective ^3^, it has been estimated that in 2013, 800,000 people sustained injuries that warranted hospital admission in the context of war, and approximately 31,000 people died as a consequence of collective violence. Despite the effects of war, military conflict has not received the same attention from public health researchers and stakeholders as many other causes of illness and death ^4^.

War is responsible for several adverse effects, and its perpetuation struggles the eradication of inequality, injustice, and exclusion. Moreover, war shatters lives, especially of those who are directly exposed to the deadly risks of the battlefield. Despite the many adverse effects of conflict, during the last decade, most epidemiological military research has been focused on describing the occurrence of combat injuries on the battlefield ^5^ ^-^ ^8^. Furthermore, efforts have been made to develop interventions aimed to improve the survival of the wounded in combat ^8^ ^,^ ^9^. However, there has been no research reported on the epidemiology of military trauma during peace negotiations. That is why our objective was to describe the variations in casualties admitted to the emergency department during the period of the negotiation of the comprehensive peace agreement in Colombia between 2011 and 2016. 

## Materials and Methods

A retrospective review of military medical patient records in Fundacion Valle del Lili (FVL) University Hospital in Cali, Colombia between January 1, 2011, and December 31, 2016. FVL is a hospital with 511 beds and serves as a referral facility for military casualties from the southwest region of Colombia. FVL is the only level I trauma center (level IV hospital in Colombia) that has a partnership agreement with the Military Forces of Colombia for the care of the soldiers wounded in combat from the Nariño, Cauca, Valle del Cauca and Southern parts of the Chocó regions (The Southwest Region of Colombia). The service area of FVL as a Level I trauma center for the wounded in combat covers almost all the southwest region of the country with an area of approximately 131,301 km^2^. The FVL institutional review board approved the study protocol (Protocol number 554).

### Patients

The FVL datasets, admission year 2011-2016, were queried for military patients with traumatic injuries by ICD-10 diagnosis codes (ICD-10 codes: Injury, poisoning and certain other consequences of external causes S00-T88). Soldiers were identified as they have army-sponsored insurance provided by the National Direction of Military Health and this characteristic is available in the medical charts and also because they are classified as "wounded in combat" at their arrival to the emergency department. We included hostile casualties (soldiers wounded in combat) ^10^. Patients whose injuries were not direct results of hostile action were excluded. Hostile casualties were defined according to the Department of Defense Dictionary of Military and Associated Terms definition ^10^as follows: "A person who is the victim of terrorist activity or who becomes a casualty <in action>. <In action> characterizes the casualty as having been the direct result of hostile action, sustained in combat or relating thereto, or sustained going to or returning from a combat mission provided that the occurrence was directly related to hostile action".

According to the comprehensive process of peace timeline, the moment of promulgation of the truce (20th November 2012) ^11^ was set as the dividing point. Therefore, the patients were divided into two groups: 1. early group before the truce (from January 2011 to December 2012) and 2. Late group after the truce: the negotiation period (from January 2013 to December 2016). 

We reviewed medical charts of each soldier who arrived to the emergency department during the study period. Data collected included age, mechanism of injury, and vital signs at presentation, shock index, Injury Severity Score (ISS), Abbreviated Injury Scale (AIS), type of surgical procedure performed, ICU admission, complications, and mortality. Description of combat casualty care statistics was performed. The definition of combat casualty care statistics was as follows ^6^ ^,^ ^12^: Deaths that occur in combat, before reaching medical care were defined as killed in action (KIA), while soldiers who survived until arrival at the medical treatment facility (MTF) were defined as wounded in action (WIA). The WIA group was the sum of three sub-groups. 1) Soldiers who died of wounds (DOW) from combat injuries after reaching medical care at an MTF. 2) Those treated and returned to duty (RTD) within 72 hours, and 3) those admitted to an MTF and survived/evacuated.

### Statistical Analysis

Descriptions of all patients were done using relative and absolute frequencies for qualitative variables. Continuous variables were reported as median with interquartile ranges. Variables of importance were compared between periods. Categorical variables were compared using the chi-square test or the Fisher exact text. Continuous variables were compared using the Wilcoxon rank-sum test. A *p* <0.05 was considered significant. All analyses were performed using STATA 14(r).

## Results

**Figure 1:**
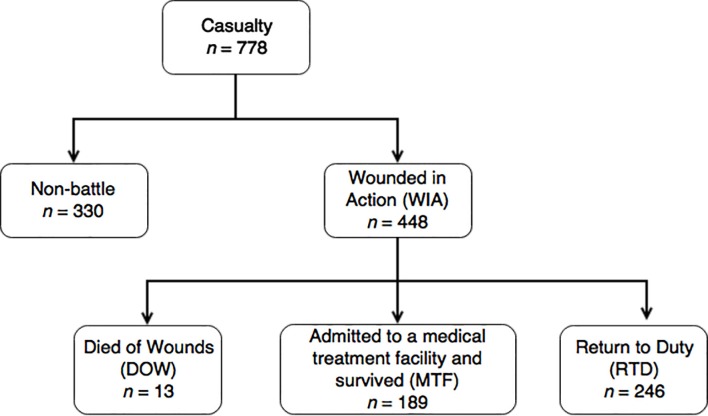
Diagram of the selection of hostile casualties.

In total, 448 WIA were recorded among 778 military patients admitted via the emergency room (ER) in the study period (2011-2016). We were not able to identify the proportion of KIA (Fig. 1). 

A description of casualty characteristics is highlighted in Table 1. All subjects were male, and three-quarters were less than 29 years old. Most of the patients (73%) were transported to the ED by helicopter. On admission, 25% of patients had a shock index greater than 0.9 and 176 (39%) had an Injury Severity Score (ISS) of 9 or higher. During the period observed, 205 (46%) patients presented with gunshot (Rifle) wounds and 222 (50%) were victims of explosions. Of these, 141 had injuries caused by antipersonnel mines. A total of 235 (52%) patients underwent emergency surgical intervention, of which 108 (24%) were admitted to ICU and 72 (16%) required damage control surgery. 

**Table 1:** Patients characteristics

	Total	Before the Negotiation Period (2011-2012)	During the Negotiation Period (2013-2016)	p-value
n	n= 448	n= 263	n= 185	
Age, median (IQR)	25 (22-29)	25 (23-29)	24 (22-28)	0.1
ISS≥9, n (%)	176 (39%)	123 (47%)	53 (29%)	<0.001
Shock Index, median (IQR)	0.7 (0.5-0.9)	0.68 (0.58-0.84)	0.69 (0.61-0.86)	0.5
Mechanism of Trauma				
Gunshot (Rifle): Injuries, n (%)	205 (45.7%)	119 (45.2%)	86 (46.4%)	0.7
Blast Injuries, n (%)	222 (49.5%)	133 (50.5%)	89 (48.1%)	0.6
Antipersonnel Mine: Injuries, n (%)	141 (31.4%)	79 (30%)	62 (33.5%)	0.4
Blunt Injuries, n (%)	7 (1.5%)	3 (1.1%)	4 (2.1%)	0.4**
Other Injuries, n (%)	14 (3.1%)	8 (3%)	6 (3.2%)	1**
Treatment of Patients				
Emergent Surgery, n (%)	235 (52%)	132 (50.1%)	103 (55.6%)	0.2
Transfusions, n (%)	98 (22%)	62 (23.5%)	36 (19.4%)	0.3
ICU admission, n (%)	108 (24%)	73 (27.7%)	35 (18.9%)	0.03
Complications, n (%)				
Enucleation, n (%)	8 (1.7%)	5 (1.9%)	3 (1.6%)	1**
Lower Extremity Amputation, n (%)	40 (8.9%)	27 (10.2%)	13 (7%)	0.3**
Spine Cord Injury, n (%)	16 (3.5%)	11 (4.1%)	5 (2.7%)	0.4**
RTD, n (%)	246 (54.9%)	143 (54.3%)	103 (55.6%)	0.7
% DOW	6.40%	5.80%	7.30%	
Mortality, n (%)	12	6	6	0.5

IQR, Interquartile Range; ISS, Injury Severity Score; ICU, Intensive Care Unit; RTD, Returned to Duty; DOW, Died of Wounds

**Fisher Exact Test

**Figure 2:**
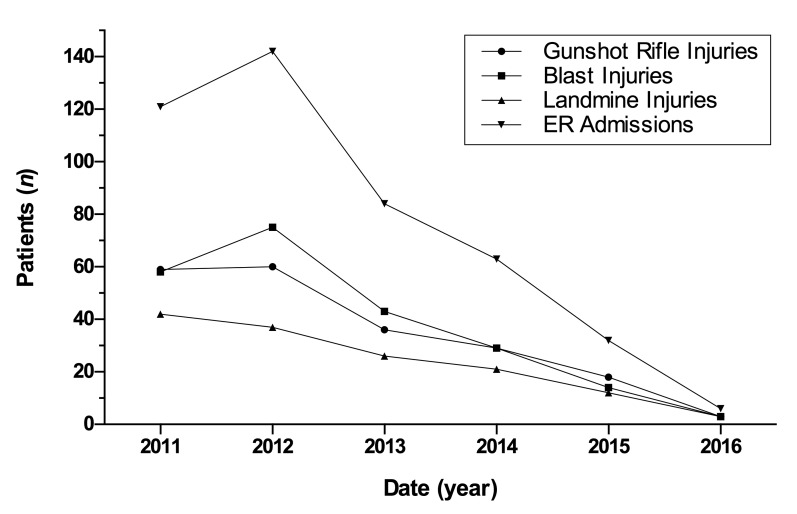
Variations in emergency department (ER) admissions of soldiers wounded in combat during the study period (cases per year). Note: landmine injuries are included in blast injuries.

Figure 2 shows the number of soldiers wounded in combat admitted to the emergency department for the years 2011 to 2016. There was a gradual decline in the emergency department admissions of hostile casualties after the beginning of the truce. Furthermore, the numbers of soldiers suffering blast and rifle injuries also decreased over this period. In 2012 there were nearly 150 hostile casualties' admissions to the ER. This number decreased to 84, 63 and 32 in 2013, 2014 and 2015, and then dropped to 6 in 2016.

The most common complication was lower extremity amputation (n= 40); followed by spinal cord injury (n= 16) and neurobehavioral deficit (n= 12). Bilateral amputation was performed in 6 patients. Other complications included enucleation (n=8) and genital mutilation (n= 2). As shown in Table 1, there was an absolute decrease in the number of complications in the late group (after the truce) with respect to the early group. Overall %DOW was 6.4%, and in-hospital mortality was 3%. Of the 448 WIA included, 55% returned to duty in the first 72 hours after reaching our MTF during the study period. 

Furthermore, we observed a gradual decrease in the number of casualties admitted to the intensive care unit, those requiring blood product transfusion and damage control surgery. (Table 2)

**Table 2:** Procedures related to trauma care and admissions to the intensive care unit per year

	Total	2011 (n= 121)	2012 (n= 142)	2013 (n= 84)	2014 (n= 63)	2015 (n= 32)	2016 (n=6)
Number of soldiers that underwent emergency surgery, n (%)	235 (100%)	68 (29%)	64 (27%)	40 (17%)	37 (16%)	21 (9%)	5 (2%)
Number of soldiers that underwent DCS, n (%)	72 (100%)	12 (17%)	14 (19%)	8 (11%)	22 (31%)	14 (19%)	2 (3%)
Number of soldiers that required transfusions of blood products, n (%)	98 (100%)	32 (33%)	30 (31%)	15 (15%)	15 (15%)	4 (4%)	2 (2%)
Number of Soldiers admitted to the ICU, n (%)	108 (100%)	37 (34%)	36 (33%)	16 (15%)	11 (10%)	6 (6%)	2 (2%)

DCS, Damage Control Surgery; ICU, Intensive Care Unit

The differences between the early group and the late group (after the truce) are highlighted in Table 1. The proportion of patients presenting with an ISS greater than or equal to 9 was significantly higher before 2013 (before the truce) (*p* >0.001). The percentage of patients admitted to ICU was also significantly greater in the early group (2011-2012) compared with the late group (after the truce) (*p*= 0.03). Finally, from August to December/2016 no admissions of war casualties were registered. 

## Discussion 

To our knowledge, this is the first known description of soldiers wounded in combat during a period of peace negotiation. We found that there was a gradual decrease in the admissions of hostile casualties to the emergency department during the period observed. Furthermore, both the performance of surgical procedures and the consequences related to trauma care also declined during the same period. This number started to fall from 2013 on forward.

Of the 448 hostile casualties identified, 263 presented before the truce (2011-2012) whereas 185 presented after the truce in the period of the negotiation and implementation of the comprehensive peace agreement (2012-2016). This number may seem low if compared with 2004 reports from Operation Iraqi Freedom and Operation Enduring Freedom, in which more than 10,000 service members suffered battlefield injuries, and almost 1,000 were killed in action ^13^. However, unlike the conflicts mentioned above, Colombian war is a political-military confrontation between government, guerrillas, and paramilitary groups, below conventional war but with the use of armed force and it can be defined as a low-intensity conflict ^14^. Although Colombian low intensity conflict is a unique combat arena, few studies have reported the characteristics of military casualties. In 2010 the subsystem of health of the Colombian Military Forces reported a total of 2,500 combat casualties; of which 500 died before reaching an MTF ^9^ ^,^ ^15^. Another study from the Hospital Militar Central in Bogotá ^9^ reporting the epidemiology of combat injuries described a total of 9,603 casualties during the period 2005-2010; of which 2,537 (26%) were killed in action. 

The current study found that the absolute number of battlefield casualties admitted to the emergency department decreased from nearly from 150 in 2012 to less than 10 in 2016. Furthermore, the proportion of patients presenting with an ISS greater than or equal to 9 was higher before the truce (2011-2012). There is a degree of certainty around the association between war and negative health outcomes. Although a generalization, it is true that war is always seen as having something to do with a rise in mortality. Several studies have demonstrated an association between war and mortality in other settings ^16^ ^-^ ^18^. For example, Li *et al* ^(^ ^18^ found that deaths during war intervals are significantly higher when compared with peace periods. Furthermore, previous studies evaluating the impact of peace on health have observed consistent results on the benefits of peace implementation on the improvement of the health of populations ^19^ ^,^ ^20^. In the context of conflict-related violence, hostile casualties can be seen as a measure of the magnitude and dangerousness of the war. Therefore, the reduction in military hostile casualties' occurrence and their consequences could be taken as a proxy indicator for the impact of peace on military trauma.

An absolute reduction in the performance of procedures related to trauma care (damage control resuscitation procedures) during the late period (2013-2016) was evident. Additionally, there was an absolute decrease in the number of complications (amputations, spinal cord injury, enucleations) in the late group (negotiation period) with respect to the early group (before the truce). Damage control resuscitation (DCR) is a structured intervention that includes early blood product transfusion, early hemorrhage control by damage control surgery (DCS) and restoration of physiologic stability ^21^ ^,^ ^22^. These interventions could have a great impact on resource utilization as patients managed following principles of DCR may require a longer intensive care unit and hospital stay and thus, higher medical resources and costs. As for complications, previous studies have investigated the impact of war on medical and economic resources ^23^ ^-^ ^25^. For example, Edwards *et al*. ^(^ ^24^,calculated the long-term cost of traumatic amputations of British personnel from Afghanistan and found that the total cost of 265 casualties that sustained a total of 416 amputations would be higher than USD 444 million over forty years. Masini *et al.* ^(^ ^25^, showed that combat-related extremity injuries require the greatest utilization of resources for inpatient treatment, are responsible for a higher burden of disabled soldiers, and have the most significant projected disability benefit costs. 

This study found that a lower number of patients were admitted to the ICU after the truce (2013-2016) when compared to the period before the truce (2011-2012). Furthermore, there was a gradual decrease in the number of casualties admitted to the ICU since the declaration of the truce. Previous studies of ICU casualty care ^26^ ^,^ ^27^ focused on the description of patients admitted to the ICU on war environments. Lundy *et al.* ^(^ ^26^, reported their one-year experience in the management of patients admitted to the ICU at the 10th Combat Support Hospital in Iraq. They found 875 patients admitted to the ICU; of which 165 (18.9%) were US soldiers. Although our methodology is not different from previous descriptive studies, this result (ICU admission) further supports the idea of the positive impact of the process of peace on military trauma. 

Although, we found a reduction in the occurrence of hostile military casualties, their severity, and their consequences probably as a result of the negotiation and implementation of the Colombian process of peace, the evolution of military care through wars history has demonstrated that other factors, different from the nation state of internal peace; such as the medical system, the improvement in trauma care and the development of new medical technologies also determines the lethality of war ^12^. However, over the study period (2011-2016) there were no significant changes in the basic principles of damage control resuscitation and surgical techniques. Therefore, it seems very unlikely that improvements in trauma care and surgical techniques were responsible for the differences found.

We acknowledge our study limitations. Firstly, before the current report, most of the studies describing the patterns of combat casualties attempted to analyze the epidemiology and outcomes of battlefield injuries on mid-intensity conflicts; such as the Operation Iraqi Freedom ^6^ ^,^ ^28^ and Operation Enduring Freedom ^5^ ^,^ ^7^ ^,^ ^13^ ^,^ ^29^. These previous reports shared the common goal of improving combat casualty care, focusing on injury prevention ^8^ ^,^ ^30^ and highlighted the importance of identifying those casualties lost on the battlefield ^5^. However, this study has been unable to analyze those deaths occurring on the battlefield as since we only had access to those wounded in action (WIA) but no to those killed in action (KIA). Secondly, despite the fact that FVL is the only level I trauma center serving as a referral facility for military casualties in the southwest region of Colombia, we can only have access to casualties who reached the emergency department. Furthermore, we did not have visibility on those from other areas of the country and those who died before reaching medical care; all of which may have introduced significant selection bias. However, other reports also have shown a reduction in the number hostile casualties and their consequences from other regions of the country probably as a result of the process of peace ^31^ ^,^ ^32^. For example, Valencia *et al.* ^(^ ^33^,showed that the number of soldiers' victims of war injuries in rural areas from Colombia decreased from 351 in 2013 to 150 in 2014. 

The methodological nature of our study does not allow us to draw strong causal inferences on the effect of peace on military casualty outcomes. However, the patients were classified based on war criteria definition injury. Additionally, there no have been others interventions aimed at stopping the civil war different from the traditional military actions regularly provided by the Colombian government. Therefore, our study suggests that a positive effect on civil war has emerged from peace negotiation.

## Conclusion

We describe a series of soldiers wounded in combat that were admitted to the emergency department before and during the negotiation of the Colombian process of peace. Overall, we found a trend toward a decrease in the number of casualties admitted to the emergency department possibly in part, as a result of the negotiation of the comprehensive process of peace. As wars are responsible for deaths and injuries on the battlefield, on-going wars must cease through the negotiation and implementation of a process of peace. 
