# Supplementary material for: Analysis of combat casualties admitted to the emergency department during the negotiation of the comprehensive Colombian process of peace
Source: Colomb Med (Cali). 2017 Dec 30;48(4):155–60. doi: 10.25100/cm.v43i4.3389 (PMC5896721; doi:10.25100/cm.v43i4.3389)
Supplement: Related texts

# Research article: Análisis de los soldados heridos en combate admitidos al departamento de emergencias durante la negociación del proceso de paz en Colombia

## Introducción

Después de décadas del conflicto armado que azotó al país, el gobierno colombiano decidió iniciar conversaciones con la guerrilla de izquierda Fuerzas Armadas Revolucionarias de Colombia (FARC), con el objetivo de terminar la violencia rural y urbana que golpeo al país por más de medio siglo. El conflicto armado en Colombia se ha caracterizado por una crisis de seguridad y humanitaria de dimensiones incalculables. En los últimos 50 años, ha habido aproximadamente 92,946 víctimas de acciones hostiles derivadas de la guerra ^1^ y 39,000 muertes violentas han ocurrido desde 1988 ^2^. 

Desde una perspectiva global ^3^, se ha estimado que en 2013 800,000 personas sufrieron heridas que requirieron cuidado hospitalario en el contexto de la guerra y aproximadamente 31,000 murieron a causa de la violencia colectiva. A pesar de los catastróficos efectos derivados de la guerra, este fenómeno no ha recibido la misma atención de parte de investigadores y tomadores de decisiones como si lo han recibo otras causas de morbilidad y mortalidad ^4^. 

Las guerras son responsables de muchos efectos deletéreos. Su perpetuación impide la erradicación de la inequidad, las injusticias y la exclusión. La guerra destruye vidas, especialmente de aquellos expuestos de manera directa a los riesgos mortíferos del campo de batalla. A pesar de todo esto, la mayoría de la investigación militar se ha enfocado en describir la ocurrencia de heridas de combate en el campo de batalla ^5^^-^^8^ y se han realizado esfuerzos para mejorar la sobrevida de aquellos que sufren heridas en combate ^8^^,^^9^. Sin embargo, no existe ningún reporte que describa la epidemiología del trauma militar durante periodos de negociación de paz. Es por esto que nuestro objetivo fue describir las variaciones en los soldados heridos en combate admitidos al departamento de emergencias durante el periodo de la negociación del proceso de paz Colombiano entre el 2011 y el 2016. 

## Materiales y Métodos

El presente reporte es una serie de casos retrospectiva. Se revisaron todas las historias clínicas de pacientes militares en el Hospital Universitario Fundación Valle del Lili (FVL) en Cali, Colombia entre el 1 de Enero del 2011 al 31 de diciembre del 2016. 

La FVL es un hospital universitario sin ánimo de lucro que cuenta con 511 camas y sirve como centro de referencia para los heridos en combate de la región suroccidental de Colombia. Este hospital es el único centro de trauma nivel I (Nivel IV en el contexto colombiano) que tiene un convenio con las fuerzas militares de Colombia para la atención y el cuidado de los soldados heridos en combate provenientes de Nariño, Cauca, Valle del cauca y la parte sur del Chocó (Región del Sur-Occidente Colombiano). El área de servicio de la FVL como un centro de trauma especializado para el cuidado de los heridos en combate cubre una gran parte de la región suroccidental con un área de aproximadamente 131,301 km^2^. 

El protocolo para este estudio fue aprobado por el comité de ética e investigación biomédica de la FVL (número de protocolo 554). 

### Pacientes

La base de datos del hospital fue revisada del 2011 al 2016. Se buscaron pacientes militares que hubieran sido víctimas de heridas traumáticas por los códigos CIE-10 relacionados con el trauma (Códigos CIE-10: S00-T88). Los soldados pudieron ser identificados ya que ellos tienen un seguro de salud exclusivo de las fuerzas militares y esta característica está disponible en las historias clínicas. Además, todos los soldados heridos en acción son clasificados como "herido en combate" una vez llegan al departamento de emergencias. 

Se incluyeron pacientes heridos en combate (soldados heridos en combate) ^10^. Los pacientes (militares) que tuvieran heridas no derivadas de acciones hostiles fueron excluidos. Los soldados heridos en combate durante acciones hostiles se definieron siguiendo la clasificación del Diccionario de términos militares del Departamento de Defensa ^10^ de la siguiente manera: "Un soldado herido en combate durante acciones hostiles es aquel que es víctima de una acción terrorista "en acción". "En acción" significa que el soldado fue víctima directa de una acción hostil durante el combate. 

Siguiendo la línea del tiempo de proceso de paz se definieron límites de tiempo. El momento de la iniciación delas conversaciones de paz y promulgación del cese al fuego unilateral por parte de las FARC (20 de noviembre 2012) ^11^ fue definido como el punto divisorio. Por lo tanto, los pacientes se dividieron en dos grupos: 1. El grupo que se presentó antes del cese al fuego unilateral (Enero 2011 a diciembre 2012) y 2. El grupo que se presentó después del cese al fuego, es decir, durante el periodo de negociación.

De las historias clínicas de los soldados heridos en combate que ingresaron al departamento de emergencia se extrajo la siguiente información: edad, mecanismo del trauma, signos vitales al ingreso, el índice de shock, el índice de severidad del trauma (ISS) y el tipo de intervención quirúrgica realizada; ingreso a la UCI, complicaciones y mortalidad. Además se calcularon estadísticas específicas del trauma militar. Las estadísticas específicas del trauma militar se definieron de la siguiente manera ^6^^,^^12^: las muertes que ocurrieron en el campo de batalla y antes de llegar a un centro médico se definieron como "muertos en acción (KIA: *Killed in Action*)". Los soldados que sobrevivieron hasta llegar a un centro médico se definieron como "heridos en acción: WIA: *Wounded in Action*)". El grupo de "heridos en acción (WIA)" fue la suma de tres subgrupos. 1. Los soldados que murieron a causa de las heridas de combate después de llegar al centro médico (DOW: *Died of Wounds*), 2. Aquellos que fueron tratados y retornaron a prestar sus servicios en las primeras 72 horas (RTD: *Returned to Duty*) y, 3. Aquellos que fueron admitidos a un centro médico y sobrevivieron (MFT: *Survived until arrival at the medical treatment facility*). 

### Análisis estadístico

Las variables cualitativas fueron presentadas usando frecuencias relativas y absolutas. Las variables continuas se reportaron usando la media y el rango intercuartílico. Las variables de importancia se compararon entre los periodos de tiempo. Las variables categóricas se compararon usando la prueba de chi-2 o el test exacto de Fisher. Las variables continuas se compararon usando el Wilcoxon Rank-sum test. Una *p* <0.05 se consideró significativa. Todos los análisis se realizaron utilizando el Software STATA versión 14. 

## Resultados

Un total de 448 soldados heridos en acción fueron incluidos entre 778 pacientes militares que ingresaron al departamento de emergencias durante el periodo estudiado (2011-2016). No pudimos identificar la proporción de los soldados muertos en acción (KIA: Killed in Action) (Fig. 1). 

**Figure 1:** Diagrama para la selección de los soldados

La Tabla 1 muestra una descripción de las características demográficas y clínicas de los soldados heridos en combate incluidos. Todos los sujetos fueron hombres y tres cuartas partes fueron menores de 29 años. La mayoría de los pacientes (73%) fueron transportados al departamento de emergencias en helicóptero. Al momento de la admisión 25% de los pacientes tenía un índice de shock mayor de 0.9 y 176 (39%) tenían un Índice de severidad del trauma (ISS) mayor o igual a 9.

**Table 1:** Características de los pacientes

	Total	Antes del periodo de negociación (2011-2012)	Durante el periodo de negociación (2013-2016)	Valor *p*
	n= 448	n= 263	n= 185	
Edad, mediana (RIC)	25 (22-29)	25 (23-29)	24 (22-28)	0.1
ISS ≥9, n (%)	176 (39%)	123 (47%)	53 (29%)	<0.001
Índice de Shock, mediana (RIC)	0.7 (0.5-0.9)	0.68 (0.58-0.84)	0.69 (0.61-0.86)	0.5
Mecanismo de Trauma				
Heridos por Fusil, n (%)	205 (45.7%)	119 (45.2%)	86 (46.4%)	0.7
Heridos por Explosión, n (%)	222 (49.5%)	133 (50.5%)	89 (48.1%)	0.6
Heridos por Minas Antipersona, n (%)	141 (31.4%)	79 (30%)	62 (33.5%)	0.4
Trauma Cerrado, n (%)	7 (1.5%)	3 (1.1%)	4 (2.1%)	0.4**
Otros, n (%)	14 (3.1%)	8 (3%)	6 (3.2%)	1**
Tratamientos				
Cirugía de Emergencia, n (%)	235 (52%)	132 (50.1%)	103 (55.6%)	0.2
Transfusiones, n (%)	98 (22%)	62 (23.5%)	36 (19.4%)	0.3
Admisiones a la UCI, n (%)	108 (24%)	73 (27.7%)	35 (18.9%)	0.03
Complicaciones, n (%)				
Enucleación, n (%)	8 (1.7%)	5 (1.9%)	3 (1.6%)	1**
Amputaciones, n (%)	40 (8.9%)	27 (10.2%)	13 (7%)	0.3**
Trauma Raquimedular, n (%)	16 (3.5%)	11 (4.1%)	5 (2.7%)	0.4**
Retornó a prestar Servicios en las primeras 72 horas (RTD), n (%)	246 (54.9%)	143 (54.3%)	103 (55.6%)	0.7
Murió a Causa de sus heridas (DOW) (%)	6.40%	5.80%	7.30%	
Mortalidad, n (%)	12	6	6	0.5

RIC, Rango Intercuartílico; ISS, Injury Severity Score; UCI, Unidad de Cuidado Intensivo RTD,

**Fisher Exact Test

Durante el periodo de estudio, 205 (46%) pacientes se presentaron con heridas por arma de fuego (fusiles) y 222 (50%) fueron víctimas de explosiones. De estos, 141 tenían heridas causadas por minas anti-persona. Un total de 235 (52%) pacientes fueron llevados a cirugía de emergencia de los que 108 (24%) fueron admitidos a la unidad de cuidado intensivo y 72 (16%) requirieron cirugía de control de daños. 

La Figura 2 muestra el número de soldados heridos en combate admitidos al departamento de emergencias del 2011 al 2016. Hubo una disminución gradual en las admisiones al departamento de emergencias después del inicio del cese al fuego. Además, el número de soldados que sufrieron heridas por fusiles y explosiones también disminuyó durante este periodo. En 2012 hubo casi 150 admisiones, este número disminuyó a 84, 63 y 32 en los años 2013, 2014 y 2015. En 2016 hubo 6 admisiones de soldados heridos en combate al departamento de emergencias. 

**Figure 2:** Variaciones en la admisión de soldados heridos en combate durante el periodo del estudio (casos por año). Nota: Las víctimas por minas antipersona se incluyen en las víctimas por explosiones.

La complicación más común fue la amputación de las extremidades inferiores (n= 40); seguida por el trauma raquimedular (n= 16) y el déficit neuro-sensorial (n= 12). Seis pacientes fueron sometidos a amputación bilateral de miembros inferiores. Otras complicaciones incluyeron enucleación (n= 8) y mutilación genital (n= 2). Como se ve en la Tabla 1, hubo una disminución absoluta en el número de complicaciones en el grupo tardío (durante el periodo de negociación) con respecto al grupo temprano (antes del cese al fuego unilateral). La proporción de soldados que murieron a causa de heridas de combate (DOW: *Died of Wounds*) fue del 6.4%, y la mortalidad hospitalaria del 3%. De los 448 soldados heridos en combate incluidos, 55% volvieron a prestar sus servicios en las primeras 72 horas (RTD: *Returned to Duty*). 

Se observó una disminución gradual en el número de soldados heridos en combate admitidos a la unidad de cuidados intensivo, aquellos que requirieron transfusiones de productos sanguíneos y cirugía de control de daños (Tabla 2). 

**Table 2:** Procedimientos y admisiones a la UCI por año

	Total	2011 (n=121)	2012 (n=142)	2013 (n=84)	2014 (n=63)	2015 (n=32)	2016 (n=6)
Número de soldados que requirieron cirugía de emergencia, n (%)	235 (100%)	68 (29%)	64 (27%)	40 (17%)	37 (16%)	21 (9%)	5 (2%)
Número de soldados que requirieron CCD, n (%)	72 (100%)	12 (17%)	14 (19%)	8 (11%)	22 (31%)	14 (19%)	2 (3%)
Número de soldados que requirieron transfusión de hemoderivados, n (%)	98 (100%)	32 (33%)	30 (31%)	15 (15%)	15 (15%)	4 (4%)	2 (2%)
Número de soldados admitidos a la UCI, n (%)	108 (100%)	37 (34%)	36 (33%)	16 (15%)	11 (10%)	6 (6%)	2 (2%)

CCD, Cirugía de Control de daños; UCI, Unidad de cuidado intensivo

Las diferencias entre los dos grupos (antes del cese al fuego unilateral vs. Durante el periodo de negociación) se muestran en la Tabla 1. La proporción de pacientes que se presentan con un ISS mayor o igual a 9 fue significativamente mayor antes del 2013 (*p* <0.001). El porcentaje de pacientes admitidos a la UCI también fue significativamente mayor durante el periodo temprano. Finalmente, de agosto a diciembre del 2016 no hubo admisiones de heridos en combate. 

## Discusión

Este estudio muestra el primer análisis conocido de soldados heridos en combate durante un periodo de negociación de paz. Encontramos que hubo una disminución gradual en la admisión de soldados heridos en combate al departamento de emergencias durante el periodo observado. Además, hubo una diminución absoluta en el número de procedimientos de reanimación y quirúrgicos relacionados con el trauma durante el mismo periodo. La tendencia descrita empezó a ser más evidente del 2013 hacia delante. 

De los 448 soldados heridos en combate identificados, 263 se presentaron antes del cese al fuego unilateral (2011-2012) mientras que 185 se presentaron después, es decir, durante el periodo de negociación. El número de soldados presentados en este reporte puede parecer bajo si se compara con reportes del 2004 de las operaciones "*Iraqi Freedom*" y "*Enduring Freedom*" en las que más de 10,000 soldados sufrieron heridas de combate y aproximadamente 1,000 murieron en acción ^13^. Sin embargo, a diferencia de lo sucedido en estos reportes, la guerra colombiana es un conflicto diferente pues se trata de una confrontación político-militar entre el gobierno, la guerrilla y los grupos paramilitares. Es un conflicto con una intensidad menor a la guerra convencional, pero en el que intervienen las fuerzas armadas y se despliegan operaciones militares y puede ser definido como un conflicto de baja intensidad ^14^. Aunque se trate de una guerra de características únicas y con un campo de batalla particular, pocos estudios han reportado las características de los heridos en el campo de batalla durante el conflicto colombiano. En el 2010, el subsistema de salud de las Fuerzas Militares de Colombia reportó un total de 2500 heridos en combate, de los cuales 500 murieron antes de alcanzar un centro médico ^9^^,^^15^. Otro estudio realizado en el Hospital Militar Central de Bogotá ^9^ reportó un total de 9603 heridos en combate de los cuales 2,537 murieron en acción para el periodo 2005-2010. 

En este estudio se encontró que el número absoluto de soldados heridos en combate admitidos al departamento de emergencias disminuyó de 150 en el 2012 a menos de 10 en el 2016. Además, la proporción que se presentó con un ISS mayor o igual a 9 fue mayor antes del periodo de negociación (2011-2012). Existe una asociación entre la guerra y la ocurrencia de desenlaces negativos en salud. Aunque parezca evidente, es cierto que los conflictos bélicos tienen influencia en el aumento de la mortalidad. Varios estudios han demostrado una asociación entre la guerra y la mortalidad en otros escenarios ^16^^-^^18^. Por ejemplo, Li *et al*. ^18^, demostraron que las muertes ocurridas durante los periodos de guerra eran significativamente mayores cuando se compararon con periodos de paz. En el mismo sentido, estudios previos que han evaluado el impacto de la paz en la salud de las poblaciones y han encontrado resultados consistentes en cuanto a los efectos benéficos de la implementación de la paz en el mejoramiento de las condiciones de salud ^19^^,^^20^. En el contexto de la violencia relacionada con el conflicto armado, el número de heridos en combate puede ser una medida indirecta de la magnitud y la peligrosidad de la guerra. Por lo tanto, la reducción en el número de heridos en combate y sus consecuencias podrían ser tomadas como medidas subrogadas del impacto de la paz en el trauma militar. 

En el presente reporte hubo también una reducción absoluta del número de procedimientos de resucitación de control de daños (transfusiones, cirugía de control de daños) durante el periodo de negociación. Igualmente, hubo una disminución en el número de desenlaces adversos como amputaciones, trauma raquimedular y enucleaciones. La resucitación de control de daños es una intervención estructurada que incluye la transfusión temprana de hemoderivados, el control precoz de la hemorragia y la restauración del estado fisiológico en la unidad de cuidado intensivo ^21^^,^^22^. Estas intervenciones podrían tener un gran impacto en la utilización de recursos ya que los pacientes manejados siguiendo los principios de la resucitación de control de daños requieren estancias prolongadas en la unidad de cuidado intensivo y en el hospital y, por lo tanto, mayores recursos médicos y económicos. En cuanto a las complicaciones, estudios previos han investigado el impacto de la guerra y sus consecuencias en los recursos médicos y económicos ^23^^-^^25^. Por ejemplo, Edwards *et al*. ^24^, calcularon el costo a largo plazo de las amputaciones traumáticas en el personal del ejército británico con presencia en Afganistán. En este estudio se describió que el costo total de 265 heridos en combate a quienes se les realizó 416 amputaciones pudo haber sido mayor de 444 millones de dólares en un periodo 40 años. Masini *et al*. ^25^, mostraron que las heridas de las extremidades derivadas del campo de batalla requirieren la mayor inversión de recursos económicos para el cuidado hospitalario y son responsables de la mayor carga de enfermedad. 

En este estudio encontramos que un número menor de pacientes fueron admitidos a la Unidad de Cuidados Intensivos durante el periodo de negociación (2013-2016). Además, hubo una disminución gradual y sostenida en el número de soldados heridos en combate admitidos a la unidad de cuidado intensivo desde la declaración del cese al fuego. Estudios previos sobre el cuidado crítico de soldados heridos en combate ^26^^,^^27^ se han enfocado en la descripción de pacientes admitidos a la UCI en ambientes hostiles y durante guerras en curso. Lundy *et al*. ^26^, reportaron su experiencia de un año en el manejo de pacientes admitidos a la UCI en el *10th Combat Support Hospital* en Iraq. En ese estudio describieron 875 pacientes admitidos, de los cuales 165 (18.9%) fueron soldados del ejército de Estados Unidos. Aunque la metodología usada en este reporte no es diferente a la de estudios previos, nuestra descripción sobre la disminución del número de ingresos a la UCI soporta la idea del impacto positivo del proceso de paz en el trauma militar. 

Nuestro estudio describe una reducción en la admisión de soldados heridos en combate al departamento de emergencias, su gravedad y sus consecuencias probablemente como resultados de la negociación y la implementación del proceso de paz. Sin embargo, la historia de la evolución del trauma militar a través de las guerras nos ha enseñado que otros factores, diferentes al estado de paz, como el mejoramiento de los sistemas de trauma y el desarrollo de nuevas tecnologías también determinan la sobrevida o letalidad de la guerra ^12^. A pesar de esto, durante el periodo observado (2011-2016) no hubo mejorías significantes en los principios del cuidado del trauma y la resucitación del control de daños. Por lo tanto, parece improbable que estos factores fueran responsables por las diferencias y la tendencia observada. 

Queremos mencionar las limitaciones de nuestro estudio. Primero, antes de este reporte la mayoría de estudios se enfocaron en analizar la epidemiología y los desenlaces de los soldados heridos en el campo de batalla en conflictos de intensidad media ^5^^-^^7^^,^^13^^,^^28^^,^^29^. Estos reportes compartían el objetivo común de mejorar la calidad del cuidado de los heridos en combate, se enfocaban en la prevención del trauma ^8^^,^^30^ y destacaban la importancia de analizar las muertes que ocurrían en el campo de batalla ^5^. A diferencia de los estudios previos, nuestro reporte no analiza las muertes ocurridas en el campo de batalla ya que solo tuvimos accesos a los soldados heridos en acción que alcanzaron a ser atendidos en nuestro departamento de emergencias. Segundo, a pesar de que la FVL es el único centro de trauma especializado que sirve como centro de referencia para los soldados heridos de la región sur-occidental de Colombia, solo tuvimos acceso a los soldados que pudieron llegar a nuestro hospital. Además, no tuvimos información de los soldados de otras áreas del país ni de los que murieron antes de llegar al departamento de emergencias. Todo esto pudo haber introducido sesgo de selección e información a nuestro análisis. Sin embargo, otros reportes de otras áreas del país han mostrado una reducción similar a la reportada en este estudio probablemente como consecuencia del periodo de negociación ^31^^,^^32^. Por ejemplo, Valencia *et al*. ^33^, describieron que el número de soldados víctimas de heridas de guerra en las áreas rurales de Colombia disminuyó de 351 en el 2013 a 150 en el 2014. 

La metodología usada en nuestro reporte no nos permite establecer relaciones causales ni hacer inferencias sobre el efecto positivo de la paz en el trauma militar. Sin embargo, los pacientes fueron clasificados siguiendo definiciones establecidas para los soldados heridos en combate. Adicionalmente, no ha habido otras intervenciones dirigidas a acabar la guerra civil diferentes a las tradicionales operaciones militares realizadas por el estado colombiano. Por lo tanto, nuestro estudio y nuestra descripción sugieren que puede haber un efecto positivo de la negociación de la paz en el trauma militar. 

## Conclusión

En este estudio se describe una serie de soldados heridos en combate que fueron admitidos al departamento de emergencias antes y durante la negociación del proceso de paz. En general, encontramos una tendencia hacia la disminución en el número de soldados admitidos al departamento de emergencias y esto se debió posiblemente a la negociación del proceso de paz. Dado que las guerras son responsables por múltiples efectos deletéreos, pero especialmente por las muertes en el campo de batalla, los conflictos en curso deberían parar sus acciones e implementar la negociación de un proceso de paz.
